# Performance Improvement with Reduced Number of Channels in Motor Imagery BCI System

**DOI:** 10.3390/s25010120

**Published:** 2024-12-28

**Authors:** Ali Özkahraman, Tamer Ölmez, Zümray Dokur

**Affiliations:** 1 Department of Electronics and Communication Engineering, Istanbul Technical University, 34467 Istanbul, Istanbul, Turkey; 2 Department of Electrical and Electronics Engineering, Iskenderun Technical University, 31200 Iskenderun, Hatay, Turkey

**Keywords:** electroencephalography classification, eeg channel reduction, eog noise, motor imagery bci system

## Abstract

Classifying Motor Imaging (MI) Electroencephalogram (EEG) signals is of vital importance for Brain–Computer Interface (BCI) systems, but challenges remain. A key challenge is to reduce the number of channels to improve flexibility, portability, and computational efficiency, especially in multi-class scenarios where more channels are needed for accurate classification. This study demonstrates that combining Electrooculogram (EOG) channels with a reduced set of EEG channels is more effective than relying on a large number of EEG channels alone. EOG channels provide useful information for MI signal classification, countering the notion that they only introduce eye-related noise. The study uses advanced deep learning techniques, including multiple 1D convolution blocks and depthwise-separable convolutions, to optimize classification accuracy. The findings in this study are tested on two datasets: dataset 1, the BCI Competition IV Dataset IIa (4-class MI), and dataset 2, the Weibo dataset (7-class MI). The performance for dataset 1, utilizing 3 EEG and 3 EOG channels (6 channels total), is of 83% accuracy, while dataset 2, with 3 EEG and 2 EOG channels (5 channels total), achieves an accuracy of 61%, demonstrating the effectiveness of the proposed channel reduction method and deep learning model.

## 1. Introduction

Motor Imagery (MI) Electroencephalogram (EEG) signals are crucial for Brain–Computer Interface (BCI) systems as they provide the data needed to interpret imagined limb movements. However, BCI systems face two main limitations: (i) performance tends to decrease as the number of classes increases, and (ii) BCI systems usually contain a large number of electrodes. The decline in performance as the number of classes increases may be attributed to the different brain regions activated by each motor imagery. As the number of classes increases, the relevant centers become closer and their differentiation becomes more difficult. Effectively distinguishing these centers using a limited number of channels is a challenging task. Taking into account the inherently noisy nature of EEG signals further complicates the issue.

Motor Imagery signals are generally obtained using EEG channels, while Electrooculogram (EOG) channels are generally used for detecting and removing eye movement artifacts. Incorporating EOG channels helps minimize the impact of eye noise on EEG data, leading to cleaner and more accurate motor imagery signal classification. This approach ensures that the detected signals are more reflective of actual neural activity rather than being contaminated by eye movements.

There are some studies focusing on the Electrooculogram (EOG) channels to reduce ocular artifacts in MI BCI systems. This study [1] develops a new normalization model utilizing one contralateral EOG channel to retain MI-related neural potential while avoiding redundant influence among the EOG channels. By applying Hjorth features, sub-optimal weights for the normalization model are learned, enhancing the accuracy of MI classification in the evaluation data. In a study [2], the lightweight linear regression model is used for eye-related artifact removal, effectively reducing noise while preserving essential neural signals for improved motor imagery classification. This study [3] focuses on an automatic artifact correction method that combines regression analysis with independent component analysis (ICA) for recovering the original source signals, effectively removing eye-related artifacts while retaining the neural information critical for accurate motor imagery classification. In a study [4], ICA is directly applied to EOG channels for the identification of artifact components, which are then removed from the EEG data. In this study [5], a novel EEG/EOG-based hybrid BCI system is designed and applied to an online target selection experiment. Gaze direction from the EOG and the event-related (de)synchronization (ERD/ERS) induced by motor imagery from the EEG are simultaneously detected as the output of the BCI system. The target selection mechanism relies on the synthesis of the gaze direction and ERD activity. In this comprehensive analysis [6], unfiltered and twelve different low-pass filtered EOG channels undergo testing with five algorithms: simple regression, least mean squares, recursive least squares, REGICA, and AIR. Statistical results suggest that a low-pass frequency between 6 and 8 Hz offers the most optimal filtering for EOG channels, minimizing bidirectional contamination and enhancing artifact removal algorithm performance.

EOG channels may also capture neural activities related to EEG classification, meaning the recorded EOG channel consists of both neural activities and artifacts such as eye movements [7]. However, studies on channel selection (reduction) in the literature often focus exclusively on selecting the best EEG channels, overlooking the potential benefits of incorporating EOG channels, which are typically considered relevant only for eye-related activities. In our last study [8], the potential of including EOG channels to improve classification performance was discovered. Initially, it was thought that adding EOG channels would merely introduce ocular noise, which deep learning systems could automatically detect and filter out, thereby improving performance. However, it is shown in this study that EOG channels also contain valuable information related to MI-related neural activities, as noted in the study [7].

In our initial study [9] on channel reduction, we applied two filtering-based techniques, namely the Rayleigh coefficient map and divergence measure, to identify five common EEG electrodes for multi-class classification of motor imagery EEG. The emphasis of that study primarily lay in the identification of the five most optimal common EEG electrodes using filtering methods. In that study, we highlighted that motor imagery is commonly observed within the dark-banded region indicated in the first figure of the same study. This current study is not aimed to select the best EEG channels, but rather to demonstrate the value of incorporating EOG channels alongside a reduced set of EEG channels to enhance classification performance and system efficiency.

Within the existing literature, there are studies addressing the reduction in channels in motor imagery EEG signals. However, a majority of these studies focus only on two-class classification problems [10,11,12,13,14,15,16,17,18,19,20,21,22,23,24,25,26,27]. Therefore, when faced with a motor imagery classification problem involving only two classes, the classification task is often simplified. This is because the activation centers of these two classes are usually widely separated from each other.

There are existing studies focusing on the implementation of filtering-based techniques [10,12,14,15,16,20,25,26,27,28] and wrapping-based techniques [11,13,17,18,19,29,30,31] for the purpose of channel selection in motor imagery EEG signals. Filtering-based techniques in the context of channel reduction involve using specific criteria or statistical measures to rank or select the most relevant channels. Wrapping-based techniques, on the other hand, are iterative approaches that explore various combinations of channels to identify the subset that optimizes classification performance. These methods systematically evaluate different combinations of channels and evaluate their impact on classification accuracy or other performance metrics. Wrapping methods are valuable when interactions between channels play an important role in classification. However, they rely on the specific classifier employed, and as a result, the optimality of the selected features may not persist if a different classifier (or a deep learning model) is utilized. While using classifier performance as a criterion for feature selection can lead to a feature subset with high accuracy on the training dataset, it may not generalize well to external datasets in certain instances [32]. In this study, feature selection methods such as mutual information, CSP patterns, channel attention mechanism, permutation importance, and random search algorithm were used. Among them, permutation importance and random search can be considered wrapper methods because they rely on the performance of the classifier to select the most relevant features.

On the other hand, the majority of studies focus on subject-dependent channel selection procedures [23,25,26,27,28,29,30,31,33,34]. While subject-dependent channel selection can achieve high performance for individual subjects, it often lacks generalizability across different users. To improve BCI systems, it is crucial to develop approaches that are not only effective for individual subjects but also provide a more generalized framework. This would enable more robust and adaptable BCI systems that can perform well across a diverse range of users and scenarios.

Deep learning systems have emerged as powerful tools for the classification of motor imagery signals. Selecting or designing effective deep learning systems plays an important role in the classification of motor imagery signals. These systems not only serve as complex classifiers but are also excellent at feature extraction. Deep learning models automatically learn and represent complex patterns within motor imagery data, eliminating the need for manual feature engineering, which can be a time-consuming process. These models also have the capacity to improve classification performance due to their ability to capture complex relationships and hierarchies in data. In the study [35], the researchers chose to use the Minimum Distance Network (MDN) instead of the Fully Connected Neural Network (FCNN). They incorporated Walsh vectors into the output layer to effectively reduce the number of nodes and parameters. This innovative method, named DivFE (Divergence-based Feature Extractor), introduces a distinctive deep learning model specifically designed to address the limitations of traditional deep learning systems. The effectiveness of their algorithm in classifying MI EEG signals was also demonstrated [35,36]. EEGNet [37] is a deep learning architecture tailored for EEG data analysis. It leverages temporal convolutional layers alongside depthwise separable convolutions to efficiently capture both temporal and spatial patterns in EEG signals. This design is particularly adept at handling the unique features of EEG data, making it a powerful tool for tasks like motor imagery classification in brain–computer interfaces. Capsule networks [38] are neural network architecture which is designed to overcome some limitations of traditional CNNs, particularly in handling hierarchical relationships and spatial hierarchies in data. Capsule networks have demonstrated promise in comprehending spatial hierarchies within complex data. Researchers have demonstrated the effectiveness of this neural network model in the classification of MI EEG signals [39].

The goal of this work is to improve or maintain performance in multi-class classification while reducing the number of channels used. By incorporating EOG channels, the aim is to enhance signal quality and reduce reliance on a large number of EEG channels. Additionally, this study demonstrates that EOG channels carry valuable information related to neural activity, rather than merely reflecting eye-related noise.

Channel selection and importance techniques are utilized to highlight the role of EOG channels in improving classification performance.

In the equations presented in this study, all variables are real-valued. Scalar variables are written in italic font, representing individual real values. Vectors and matrices are written in bold to indicate their multidimensional nature.

## 2. Methodologies

In this study, the BCI Competition IV (2008) Dataset IIa [40,41] (dataset 1) and the Weibo dataset [42] (dataset 2) are used. BCI Competition IV Dataset IIa (dataset 1) contains data from nine subjects, each performing motor imagery tasks across four distinct classes: left hand, right hand, both feet and tongue. The data were recorded at a sampling rate of 250 Hz using 22 EEG and 3 EOG channels. Each participant completed two sessions on separate days, with each session comprising six runs and 48 trials per run, resulting in a total of 576 trials per subject across both sessions. During the trials, participants sat comfortably in front of a computer screen. Each trial began with a fixation cross displayed on a black screen, accompanied by a short auditory warning signal. After two seconds, an arrow appeared on the screen for 1.25 s, indicating the motor imagery task to perform. Participants were instructed to maintain the motor imagery task until the fixation cross disappeared at six seconds, followed by a short rest period where the screen remained blank. In both subject-dependent and subject-independent experiments, 80% of the dataset was allocated for training and the remaining 20% for testing.

The Weibo dataset (dataset 2) was utilized to explore the distinctions in EEG patterns between simple limb motor imagery and compound limb motor imagery tasks. For this dataset, ten subjects performed seven motor imagery tasks, including combined motor imagery tasks: left hand, right hand, feet, hands, rest, left hand with right foot, and right hand with left foot. The data were sampled at a frequency of 200 Hz, utilizing 60 EEG channels and 2 EOG channels. Data collection for each participant took place in a single session, which included 80 trials for each of the seven classes, resulting in a total of 560 trials. Each trial lasted 8 s, starting with a white circle appearing at the center of the screen. After 2 s, a red circle (preparation cue) appeared for 1 s to prepare the subjects for the upcoming task. Subsequently, the character indicating the motor imagery task (‘Left Hand’, ‘Left Hand & Right Foot’, etc.) was displayed for 4 s, during which participants were instructed to perform kinesthetic motor imagery while avoiding any muscle movement. At the 7 s mark, the word ‘Rest’ appeared for 1 s before the next trial began. In both subject-dependent and subject-independent scenarios, 80% of the dataset is allocated for training, with the remaining 20% reserved for testing across all trials.

EEG signals have some unique characteristics. They exhibit different frequency bands such as alpha, beta, theta, and delta waves, each associated with different cognitive states and activities. Table 1 shows the frequency range and cognitive states for each frequency band of EEG signals. In addition, EEG signals show a 1/f characteristic; here power decreases as frequency increases, reflecting the broad, self-similar nature of neural activity.

**Figure 1:**
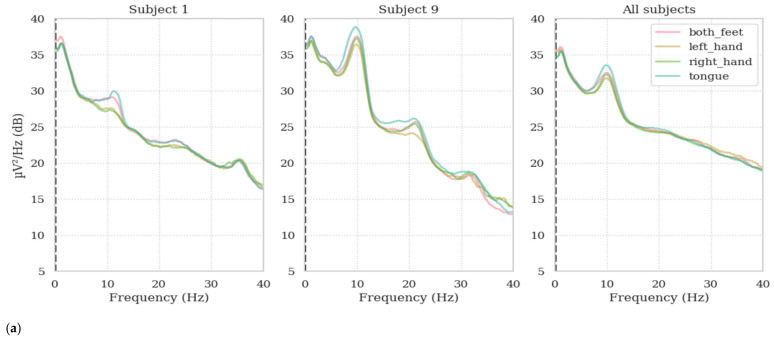

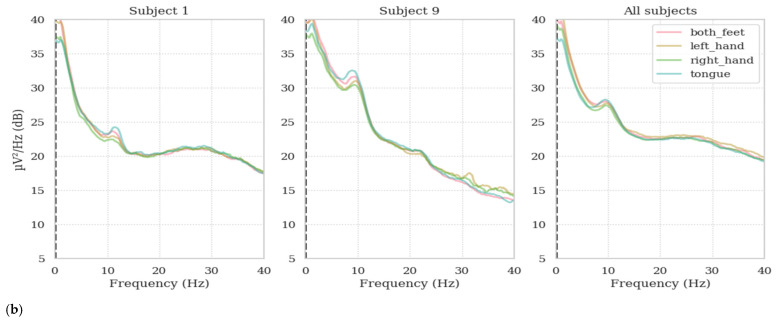
The average PSDs belonging to dataset 1 for Subject 1, Subject 9, and all subjects; (**a**) 22 EEG channels; (**b**) 3 EOG channels.

**Figure 2:**
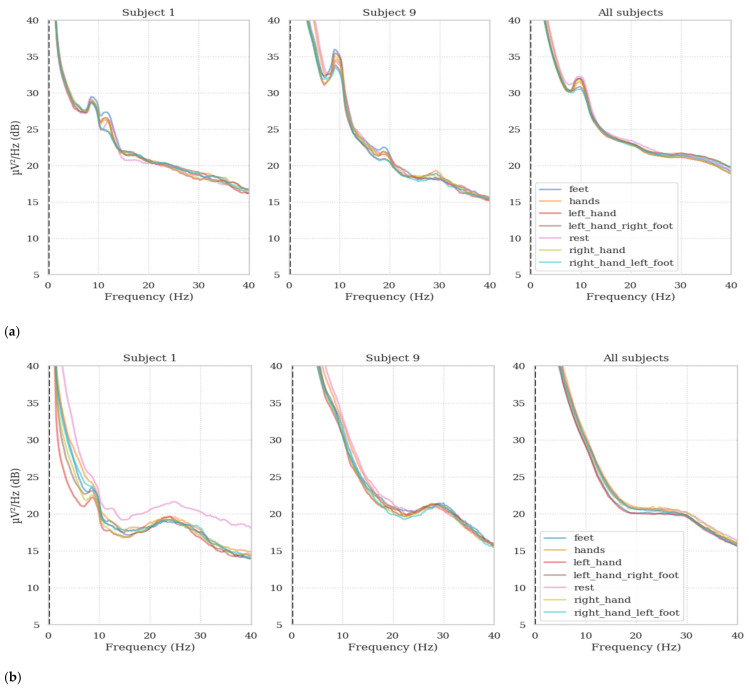
The average PSDs belonging to dataset 2 for Subject 1, Subject 9, and all subjects; (**a**) 60 EEG channels; (**b**) 2 EOG channels.

In Figure 1 and Figure 2, the power spectral density (PSD) plots for each class, for both EEG and EOG channels, are shown for different subjects (Subjects 1 and 9), as well as for all subjects combined, using data from both dataset 1 and dataset 2, respectively. The x-axis represents the frequency, ranging from 0.1 to 40 Hz, while the y-axis represents the power spectral density in units of μV^2^/Hz, expressed in decibels (dB). To create Figure 1 and Figure 2, a multitaper method is applied for spectral estimation, focusing on the frequency range between 0.1 and 40 Hz. The multitaper method, known for providing smoother spectral estimates, is selected to accurately capture the power spectral density (PSD) for each class, each subject, and for all subjects combined, across both datasets. Figure 1 and Figure 2 show that the PSDs of both EEG and EOG channels preserve the 1/f properties for both datasets. This suggests that there is a consistent frequency distribution pattern across channels, indicating that EOG channels may carry similar information to EEG channels. Moreover, the class-based relationship in EOG channels is almost the same as in EEG, especially in dataset 1; this highlights a possible overlap in the information conveyed by these channels. These observations reinforce the hypothesis that EOG channels can reflect task-related neural activity. Additionally, the similarity in class-based patterns suggests that EOG channels might be capturing a mixture of neural and ocular signals relevant to the classification process.

In this study, the channel selection methods used can be categorized into different types. For example, mutual information and CSP patterns are filter-based methods. These methods analyze the data to identify important features and filter out unnecessary channels. However, they can be more sensitive to noise, as they do not consider the performance of a downstream model during the selection process. On the other hand, methods like permutation importance and random search are wrapper-based approaches. These methods evaluate the performance of a model to optimize channel selection, which can make them more robust against noise, as they select channels based on their impact on model performance. Additionally, the channel attention mechanism is more of an embedding-based method, where the model learns the importance of each channel. By using attention mechanisms, this method can dynamically highlight important channels, making it more adaptive and potentially more effective at handling complex, noisy data.

In this study, the data from all subjects for each dataset were first combined. Then, the proposed channel selection algorithms were applied to these data, providing insights into the importance of EOG channels. Afterward, a few selected EEG channels were combined with the existing EOG channels, and separate results were observed for each subject.

### 2.1. Proposed Deep Learning Model

**Figure 3:**
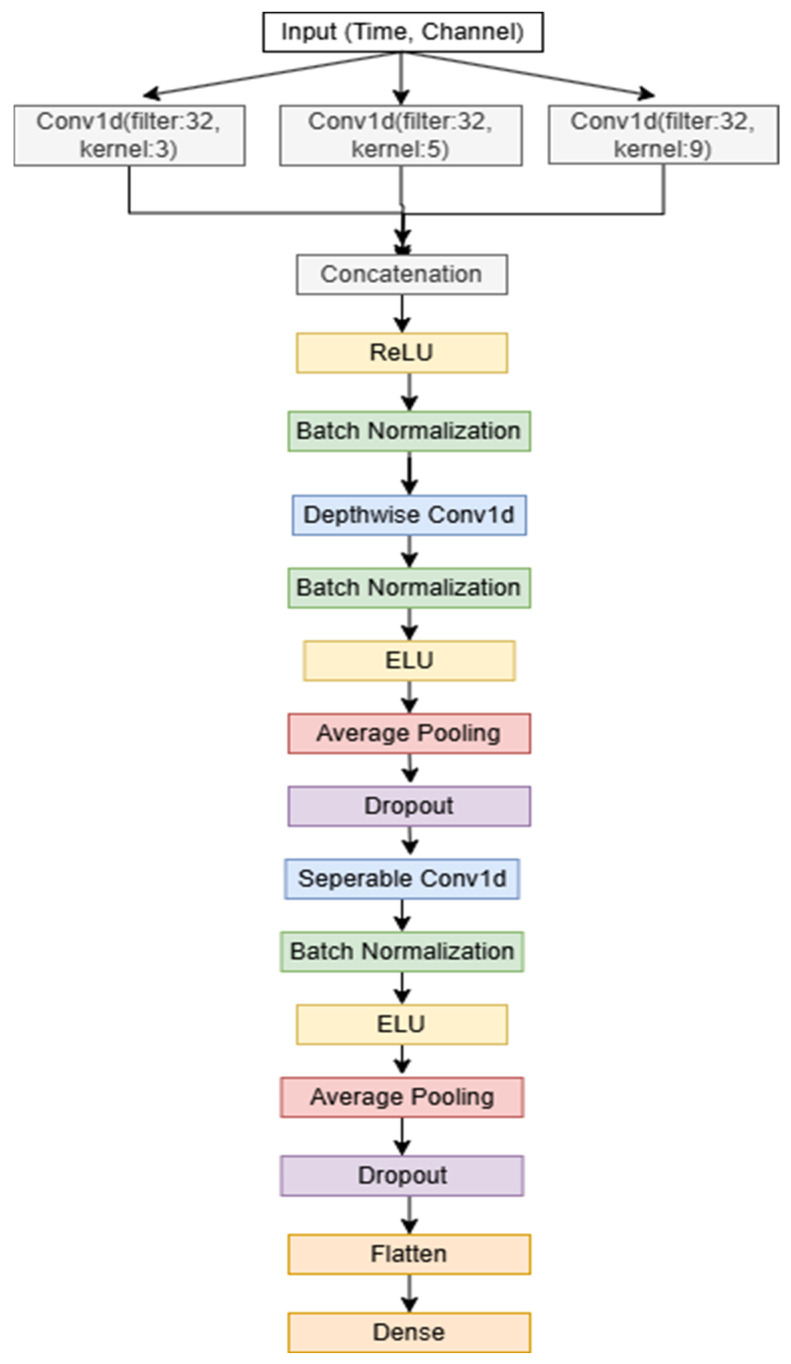
The proposed deep learning model.

The proposed model in this study utilizes the same architecture as in our previous work [8], which includes 1D depthwise and separable convolutions following the temporal convolution block. The diagram for this proposed model is shown in Figure 3. This EEGNet [37] inspired design prefers 1D convolutions over 2D convolutions to avoid a significant reduction in dimensionality. By selecting 1D convolutions, the model can preserve a higher degree of original temporal resolution in the data. This choice is crucial to preserving detailed temporal information and preventing the loss of valuable features that can occur with more aggressive dimensionality reduction methods. Ultimately, this approach helps preserve critical temporal features necessary for the accurate classification of motor imagery signals. Additionally, the model uses multiple kernel sizes within the temporal convolution block to improve feature extraction. Three kernel sizes—3, 5, and 9—were selected for the temporal convolution block, with the optimal sizes determined through trial and error. Each of these layers uses 32 filters, leading to a total of 96 output channels after concatenating the feature maps from the three layers. Smaller kernels are used to detect localized features, while larger kernels capture broader, global features. This multi-scale approach improves the model’s ability to effectively extract and analyze temporal features.

The depthwise convolution uses a kernel size of 25 and a padding of 7. The key feature of this layer is that it performs filtering independently for each input channel (with 96 input channels). This ensures that each channel receives its own filter and produces a separate output feature map for each input channel. Depthwise convolutions are used to reduce the number of parameters by applying separate filters to each input channel independently, thus creating multiple feature maps. This technique is particularly useful for datasets with multiple channels, as it prevents filter weights from being shared across channels and provides effective spatial filtering. The feature maps produced are then combined to create the final output. In the Depthwise Separable Convolution, the depthwise convolution operates as described earlier, applying a kernel size of 25 with padding of 7 and a group size of 96 (matching the input channels). This allows the model to independently filter each input channel, reducing the parameter count and focusing on channel-specific features. Separable convolution combines depthwise convolution with pointwise (1 × 1) convolution, allowing the model to first capture spatial patterns within individual channels and then efficiently integrate these patterns across channels. Using these advanced techniques, the model improves its ability to recognize complex patterns by effectively capturing both spatial and temporal characteristics of the input data.

After concatenating the results from the temporal convolutions, ReLU and batch normalization are applied. Subsequently, following the depthwise and separable convolutions, batch normalization, ELU, average pooling, and dropout layers are utilized. Batch normalization is used to standardize the output from the previous layer, which accelerates the training process and improves the model’s ability to generalize. ReLU and ELU activation functions are incorporated to introduce non-linearity into the model, allowing it to capture more complex features.

Average pooling reduces the spatial dimensions of the input tensors by averaging the values within a specified window, which helps highlight the most important features while minimizing the number of parameters. Dropout is employed to mitigate overfitting by randomly disabling a fraction of the input units during each training update, along with their associated connections. Finally, a flattened layer converts the multi-dimensional data into a one-dimensional array, followed by a dense layer used for classification.

### 2.2. Mutual Information

Mutual information (MI) is a metric used to measure the amount of information shared between two variables. In the context of analyzing EOG and EEG channels, it helps evaluate how much information can be extracted about one type of signal from another. By evaluating the common information between EOG and EEG channels, the degree of overlap in the information they provide can be determined. This is particularly useful for understanding whether EOG channels provide additional valuable information about brain activity or are merely redundant. The high mutual information between these channels indicates that EEG and EOG channels may capture overlapping information, which may contribute to the overall effectiveness of EEG-based analysis. Conversely, low mutual information may suggest that EOG channels do not contribute significant overlapping information beyond what is captured by EEG signals. The mutual information *I*(**X**;**Y**) between two random variables **X** and **Y** is defined as follows:

(1) $$I\left(X;Y\right)=H\left(X\right)+H\left(Y\right)-H(X,Y)$$

In Equation (1), *H*(**X**) is the entropy of **X**, H(**Y**) is the entropy of **Y**, and *H*(**X**,**Y**) is the joint entropy of **X** and **Y**. Entropy represents the uncertainty in the signal and can be calculated as Equation (2);

(2) $$H\left(X\right)=-\sum_{x\in X}\;P(x)loglog\;P(x)\;$$

In Equation (2), *P*(**X**) is the probability distribution of the variable **X** and *x* represents all possible values of **X**. On the other hand, the joint entropy measures the combined uncertainty of two random variables. The joint entropy between **X** and **Y** is as follows:

(3) $$H\left(X,Y\right)=-\sum_{x\in X,\;y\in Y}\;P(x,y)loglog\;P(x,y)\;$$

In Equation (3), *P*(*x*,*y*) is the joint probability distribution of **X** and **Y**.

### 2.3. Common Spatial Pattern (CSP)

CSP is employed for noise reduction, dimensionality reduction, and source separation in signal processing. It works by enhancing the contrast within the data, separating correlated components from uncorrelated ones [43]. The spatial filters used in CSP are specifically designed to maximize the difference between two features within the same dataset—one feature is enhanced to increase contrast, while the other serves as a reference [43]. These features might correspond to an experimental condition versus a control condition, or two different experimental conditions, such as left-hand MI versus right-hand MI. As a result, CSP is recognized as a supervised spatial filtering technique. Equation (4) [43] is shown for CSP as follows:

(4) $$arg\frac{w^{T}Sw}{w^{T}Rw}$$

where **w** represents the spatial filter that maximizes the equation and forms a matrix of spatial filters with dimensions R^CxC^, where C represents the number of channels. **S** and **R** are the covariance matrices with dimensions CxC corresponding to the data from class 1 and class 2, respectively. Equation (4) can be expressed as follows:

(5) $$\Lambda ={\left(w^{T}Rw\right)}^{-1}w^{T}Sw$$

In Equation (5) [43], each column in **w** denotes a spatial filter and Λ indicates the ratio in the direction of that column. By rearranging Equation (5), Equation (6) [43] can be obtained:

(6) RwΛ=Sw

Equation (6) can be resolved through generalized eigenvalue decomposition, where the eigenvectors represent the spatial filters **w** that enhance contrast, and the eigenvalues correspond to their respective weights. These calculated eigenvectors **w** are then applied to the original data to generate spatially filtered outputs.

### 2.4. Channel Attention Mechanism

The channel attention (CA) mechanism is used to detect the channels that contribute most to the classification of motor imagery signals. Unlike Mutual Information and CSP, which are considered filtering methods, the CA mechanism is a wrapper-based method that uses classifier performance to identify the most effective channels. The CA mechanism works by assigning different weights to each channel depending on its relevance to the classification task. During training, the model learns to focus more on the channels that carry the most important information, effectively improving the model’s ability to discriminate between different motor imaging tasks. This dynamic weighting allows the CA mechanism to adaptively select the best channels, optimizing the classification process.

**Figure 4:**
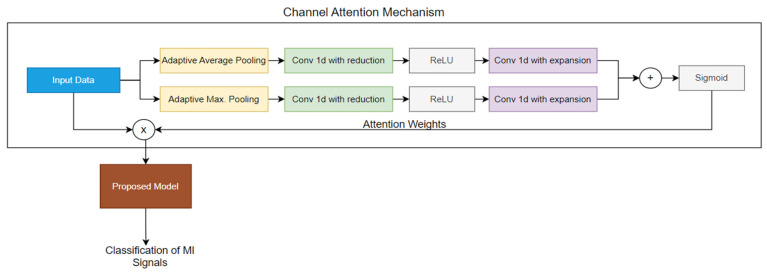
Proposed channel attention mechanism.

The proposed channel attention mechanism is shown in Figure 4. In the proposed channel attention mechanism, when the input data enters, it first undergoes two types of pooling operations: Adaptive Average Pooling and Adaptive Max Pooling. These operations summarize the information across the time dimension for each channel, capturing both the average and the most prominent features.

The pooled outputs are then passed through two 1D convolutional layers, where the first layer reduces the number of channels, and the second restores them to their original count. This approach allows the model to focus on the most important features by compressing and then restoring the channel information, enhancing the effectiveness of the attention mechanism. A ReLU activation function is applied between these layers to introduce non-linearity. The outputs from the average and max pooling paths are combined and passed through a Sigmoid activation function, which scales the output values to a range between 0 and 1. This scaling acts as a gating mechanism, determining the importance of each channel. The scaled values (attention weights) are then multiplied with the original input data, effectively amplifying the most informative channels and attenuating the less relevant ones. These refined data are then fed into the proposed deep learning model, allowing it to focus on the most significant channels and improving its ability to classify motor imagery tasks by filtering out noise and irrelevant information. This channel attention mechanism is incorporated into the model architecture and is placed between the input and the proposed deep learning model (described in Section 2.1), as shown in Figure 4.

### 2.5. Permutation Importance

Permutation Importance is another wrapper-based channel selection method used to evaluate the importance of channels (or features) in a machine learning model. It determines how much each channel contributes to the model’s predictions by measuring the change in model performance when a channel’s values are randomly shuffled. This method breaks down the relationship between the channel and the target variable, revealing how dependent the model’s predictions are on that specific channel. A significant drop in performance indicates that the channel plays a very important role, while a smaller impact indicates that the channel is less important. This approach provides valuable information about which channels are most effective, aids channel selection, and increases model interpretability.

### 2.6. Random Search

Introducing randomness into the channel selection process can improve performance as it allows for broader exploration beyond predefined hierarchies, potentially leading to better results [44]. In this method, k channels are randomly selected with equal probability, and the model is trained using these randomly chosen channels, with the accuracy serving as the weights assigned to the channels within that specific subset. This process is repeated many times and then the mean value of each channel is calculated. As a result, channels with higher scores are considered more informative and lead to the selection of the desired channels based on these scores. The calculation of scores for each channel is illustrated in Equation (7):

(7) $$c_{i}=\frac{\sum_{j=1}^{k}\;w_{j}}{k}$$

where C represents the scores of each channel, w is the accuracy of each subset, and k is the number of occurrences of that particular channel.

## 3. Experimental Results

To assess the importance and usefulness of EOG channels, this section focuses on identifying optimal channels that are consistent across all subjects. While these algorithms were introduced in Section 2, this section delves into the specific outcomes and results they provide. The analysis begins by concatenating the data from all subjects in each dataset to assess the significance of the EOG channels. These selected channels are then used in subject-wise classification to compare the performances with other studies, highlighting the significance of incorporating EOG channels.

### 3.1. Channel Importance (Selectıon) Experıments

#### 3.1.1. Mutual Informatıon

**Figure 5:**
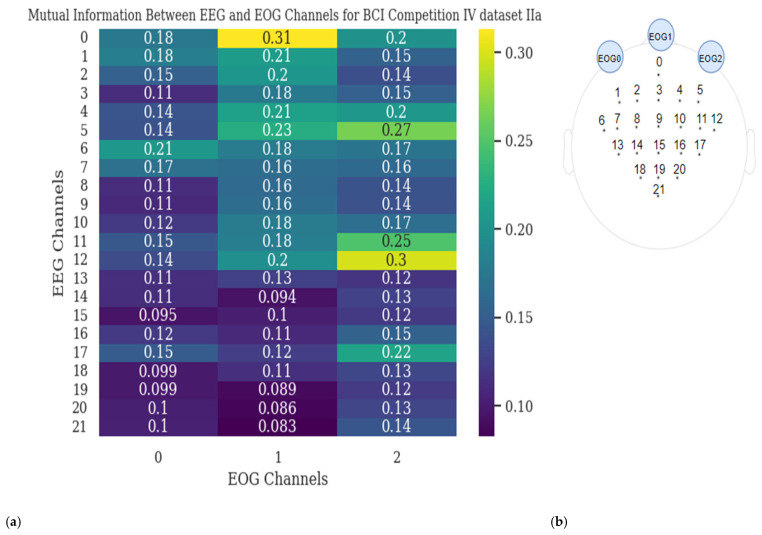
(**a**) Mutual information between EEG and EOG channels for dataset 1. (**b**) Channel locations for dataset 1.

**Figure 6:**
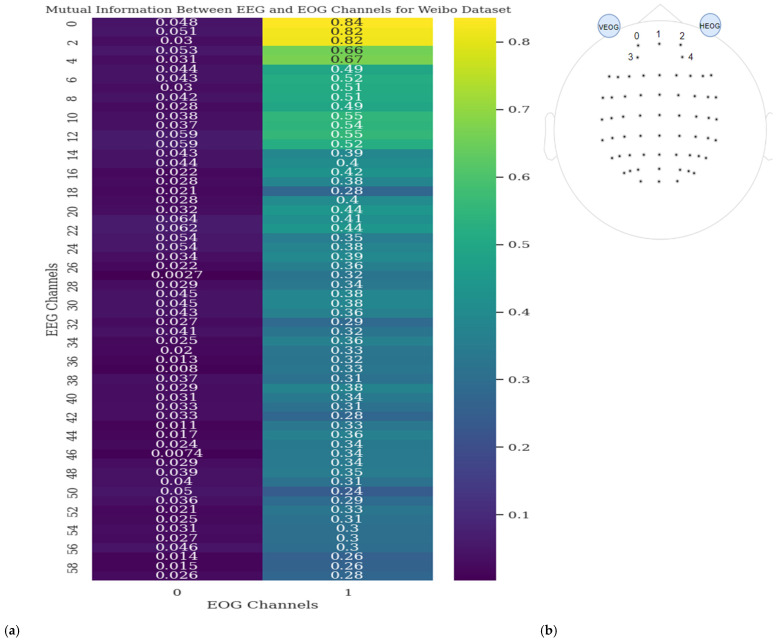
(**a**) Mutual information between EEG and EOG channels for dataset 2. (**b**) Channel locations for dataset 2.

The mutual information between EEG and EOG channels and channel locations are shown in Figure 5 for dataset 1 and in Figure 6 for dataset 2. As shown in Figure 5, EOG 0 exhibits the highest mutual information with EEG6, EEG0, and EEG1, respectively. For EOG 1, the highest mutual information is with EEG0, EEG5, EEG4 and EEG1, respectively.

Similarly, EOG 2 has the highest mutual information with EEG12, EEG5, and EEG11, respectively. It is observed that the highest mutual information between EOG and EEG channels typically occurs between channels located on the same side of the brain. In Figure 6, the Vertical Electrooculogram (VEOG) channel (index 0) exhibits low mutual information with all EEG channels, whereas the Horizontal Electrooculogram (HEOG) channel (index 1) shows high mutual information, particularly with frontal EEG channels. This suggests that these channels are likely capturing similar information, and the closer the EEG and EOG channels are to each other, the higher the mutual information score (except VEOG in dataset 2).

This proximity implies that both scenarios can occur either separately or together. It also indicates that EOG channels might be providing valuable information that complements the EEG signals. These channels located closer to the eyes highlight that both eye-related artifacts and useful information can be present, which underscores the importance of carefully analyzing and leveraging EOG channels in conjunction with EEG channels.

In the Mutual Information analysis, the aim is to highlight the significance of EOG channels by demonstrating that EEG and EOG channels may carry similar information. This similarity suggests that incorporating EOG channels could enhance the classification of motor imagery signals, as they may capture complementary or overlapping features.

Additionally, the analysis raises the possibility that EOG channels, often considered noise, could provide useful information when combined with EEG data, potentially aiding in more refined signal interpretation.

#### 3.1.2. Common Spatıal Pattern (CSP)

CSP filters are obtained at the end of the CSP process defined in Section 2.2. They are crafted to boost the separation between classes by magnifying the variance of one class and reducing that of the other within motor imagery data. These filters play an important role in altering the original EEG signals, generating a transformed space where information linked to class distinctions is amplified, and facilitating more straightforward classification. On the flip side, CSP patterns denote the spatial configurations that align with the CSP filters. These patterns depict how neural activity is distributed across various EEG channels. They provide insights into the specific regions of the brain that are most relevant to the task at hand. Therefore, the patterns provide us with more information about the appropriate channel selection procedure. The patterns provide insight into the manner in which the EEG data were generated through the distinctive neural sources extracted by the filters. This situation is also emphasized by the study [45].

**Figure 7:**
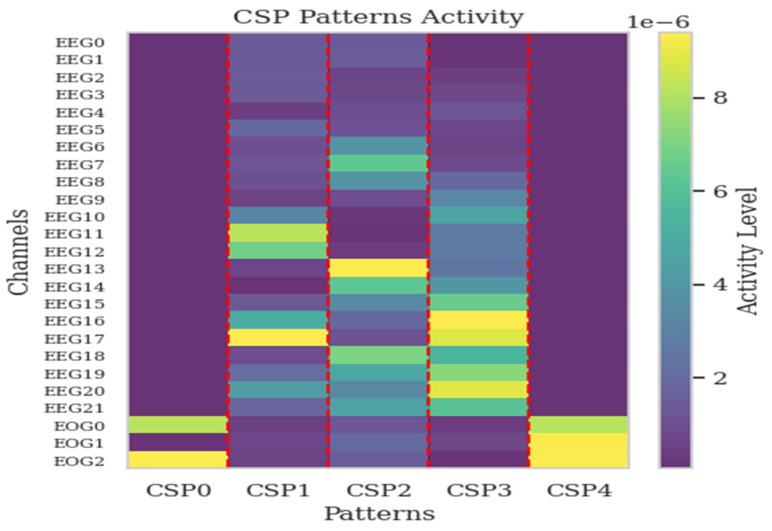
Activation of the first five spatial patterns across all channels in dataset 1.

**Figure 8:**
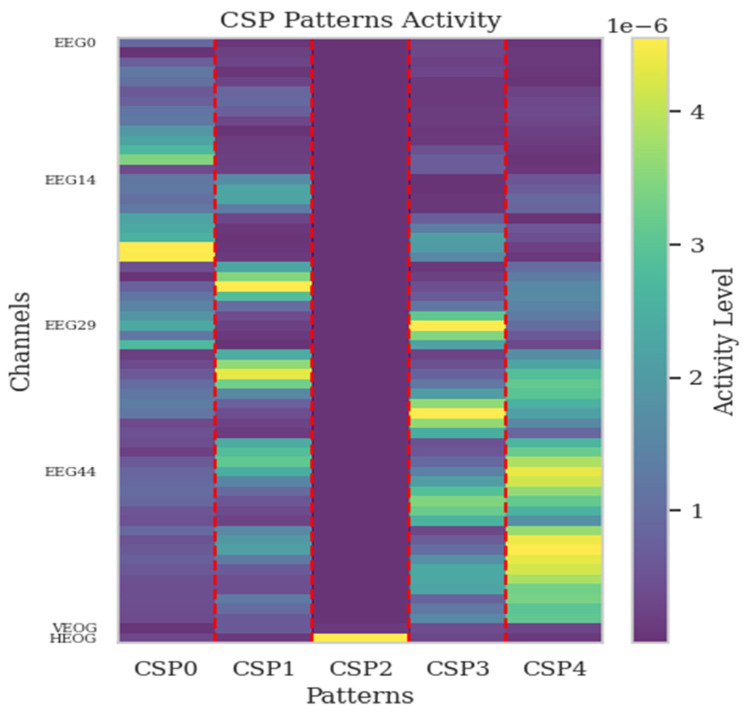
Activation of the first five spatial patterns across all channels in dataset 2.

The general CSP procedure can be defined as shown in Equation (8):

(8) $$CSP\_TransformedData=w^{T}\times OriginalData$$

where w is a matrix of spatial filters, with dimensions R^CxC^, where C represents the number of channels. *OriginalData* and *CSP_TransformedData* refer to the original and transformed data with dimensions channels x time points, respectively. Equation (8) can be written as follows:

(9) $$OriginalData={\left(w^{-1}\right)}^{T}\times CSP\_TransformedData$$

where (**w**^−1^)^T^ is a matrix of spatial patterns in Equation (9). Before obtaining these patterns, a bandpass filter is applied between 7 and 35 Hz, as motor imagery activation typically occurs within this frequency range. The initial CSP patterns capture the highest amount of information, reflecting the contribution of each channel. These patterns are particularly crucial as they highlight the most informative channels, which play a significant role in distinguishing between different motor imagery tasks. Therefore, the first spatial patterns are examined to detect the potential inclusion of EOG channels as contributing channels. By focusing on these initial patterns, the analysis aims to determine whether EOG channels play a significant role in capturing relevant neural activity. The activations corresponding to the first five spatial patterns for each channel are plotted in Figure 7 for dataset 1 and in Figure 8 for dataset 2. For dataset 1, Channels 22, 23, and 24 (last three channels as EOG0, EOG1, EOG2) are EOG channels, while for dataset 2, Channels 60 and 61 (last two channels as VEOG, HEOG) are the EOG channels.

For dataset 1, the first spatial pattern, CSP0, which is the most informative, shows that Channels 24 and 22 (EOG 2 and EOG 0) exhibit the highest activity among all channels. Additionally, CSP4 also highlights these EOG channels, with Channels 24, 23, and 22 (EOG 2, EOG 1, and EOG 0), respectively, displaying significant activity. For dataset 2, the third most important pattern (CSP2) shows that Channel 61, which is HEOG, exhibits the highest activity. Channel 60, which is VEOG, while not as active as Channel 61, ranks second in terms of activity for CSP2.

CSP is applied only during the channel selection (or importance) step, not as a general preprocessing step. These patterns obtained through CSP demonstrate the importance of including EOG channels, as they highlight that EOG channels often carry significant information such as many EEG channels. This indicates that EOG channels play a critical role in capturing relevant features for EEG classification tasks, further supporting their integration alongside EEG data for improved analysis. By focusing on these EOG channels, the model can leverage additional features that might be overlooked if only EEG data were used. Thus, incorporating EOG channels can lead to more nuanced and accurate interpretations of EEG signals.

#### 3.1.3. Permutatıon Importance

**Figure 9:**
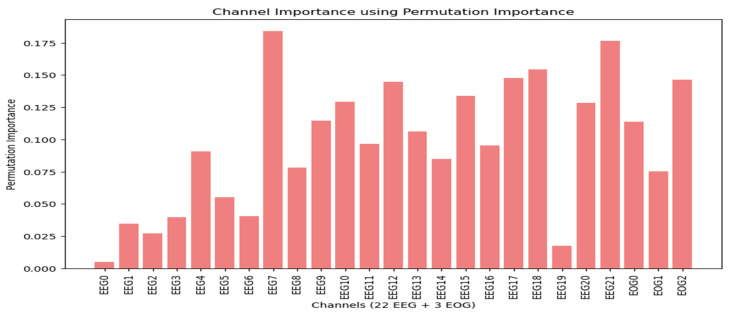
Permutation importance scores across all channels in dataset 1.

**Figure 10:**
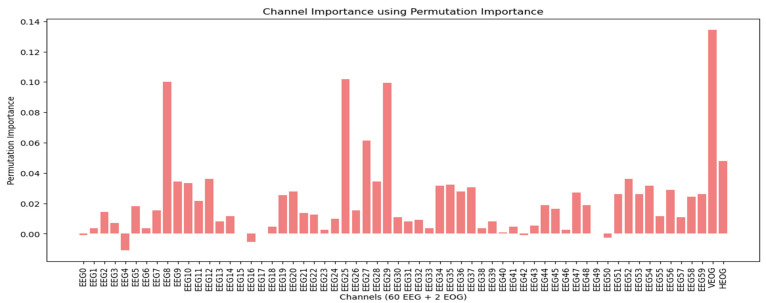
Permutation importance scores across all channels in dataset 2.

The permutation importance scores for each channel are displayed in Figure 9 for dataset 1 and in Figure 10 for dataset 2. For dataset 1, the analysis reveals that the EOG channels, particularly the last one (EOG 2), exhibit high permutation importance. EOG 0 and EOG 1 also show higher permutation importance scores compared to many of the EEG channels, indicating their significant role in the classification process.

In dataset 2, the VEOG channel stands out with the highest permutation importance score. Additionally, the HEOG channel also demonstrates a higher permutation importance score than most EEG channels. These findings underscore the substantial influence of EOG channels on classification tasks, suggesting that variations in these channels contribute significantly to the model’s performance.

#### 3.1.4. Channel Attentıon Mechanısm

**Figure 11:**
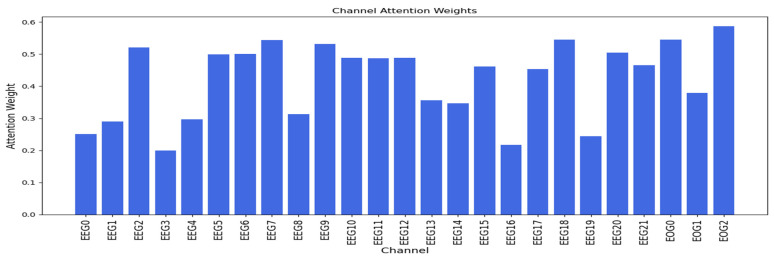
Channel attention weights across all channels in dataset 1.

**Figure 12:**
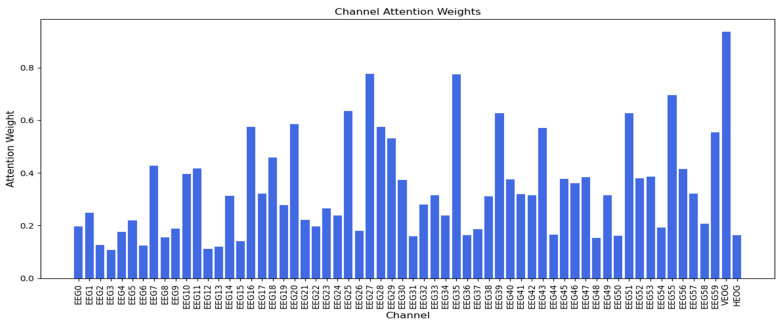
Channel attention weights across all channels in dataset 2.

Figure 11 and Figure 12 show the attention weights for dataset 1 and dataset 2, respectively. In Figure 11, the EOG 2 and EOG 0 channels have the highest attention weights among all 25 channels, with EOG 1 also having a higher attention weight than a high number of the EEG channels. In Figure 12, the VEOG channel (channel 60) has the highest attention weight, while the HEOG channel (channel 61) has an attention weight comparable to EEG channels with lower attention weights. These findings highlight the significance of EOG channels in the channel attention mechanism for both datasets, with the exception of HEOG in dataset 2.

#### 3.1.5. Random Search

The random search algorithm is applied only to dataset 1, as dataset 2 has a high number of EEG channels. Applying random search to dataset 2 would require a large number of iterations to ensure each channel is selected multiple times. Six channels were randomly selected from the total 25 channels 500 times. The accuracy obtained in each iteration serves as the weight assigned to the channels within that subset, and the mean accuracy for each channel was calculated.

**Figure 13:**
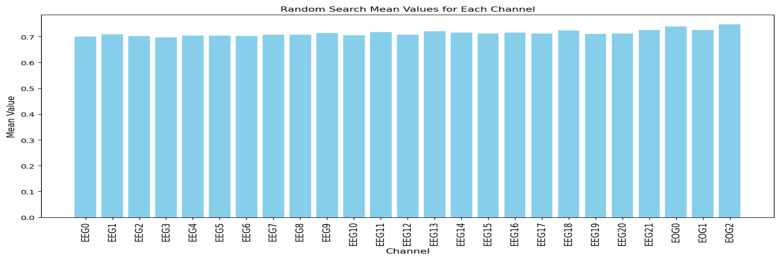
The channel performances of the random search algorithm in dataset 1.

The selection of six channels will be discussed in Section 3.2. Figure 13 shows the average channel performance for each channel in dataset 1 when using the random search algorithm. As shown in Figure 13, all channels exhibit similar activation values, but the EOG channels, particularly EOG 0 and EOG 2, demonstrate slightly better performance than all EEG channels.

#### 3.1.6. Channel Importance for Each Method

Table 2 presents the best-performing channels for four methods—CSP, Permutation Importance, Channel Attention, and Random Search—applied to dataset 1 and dataset 2, respectively. This table summarizes the experimental results obtained from the methods described above. As shown in Section 3.1.1, EOG channels (except VEOG in dataset 2) have high mutual information with EEG channels. The aim of the Mutual Information analysis is to demonstrate that both EEG and EOG can carry information for classification tasks, so it is not included in Table 2. Since the random search algorithm was not applied to dataset 2, the corresponding section in Table 2 is left blank.

As shown in Table 2, EOG channels are consistently included across all methods, illustrating their importance in the analysis of EEG signals. Their recurrent presence in the different approaches highlights the significance of incorporating EOG channels when analyzing neural activity, suggesting that these channels play a role in EEG studies. The inclusion of EOG channels across various channel selection methods points to their relevance in capturing complex signal patterns. This finding suggests that EOG channels, while often associated with eye movement, may offer insights that are integral to understanding overall signal dynamics.

### 3.2. Subject-Wıse Classıfıcatıon Accuracy by Usıng the Proposed Model

Motor imagery activity predominantly appears within the dark gray area of the first figure of the study [9], which also highlights the central regions for the four classes (left hand, right hand, tongue, and feet). Since dataset 1 includes these four classes, and dataset 2 includes both these four classes and additional combined MI classes, the activation centers for these combined MI classes are likely to be located near the activation centers of the original classes.

To represent the central regions for each MI class, one channel is chosen from the left (channel ‘C3’), one from the center (‘Cz’), and one from the right area (‘C4′) of this dark gray region for both datasets. Consequently, six channels (three EEG + three EOG) are chosen for dataset 1, and five channels (three EEG + two EOG) for dataset 2 in subject-wise classification to assess the performance of a reduced number of channels while incorporating EOG channels. To ensure compatibility, six random channels are selected using the random search algorithm for dataset 1 in Section 3.1.5. Table 3 and Table 4 present the results for dataset 1 and dataset 2, respectively. The mean, median, and standard deviation for all results across the subjects are included in Table 3 and Table 4 to provide a comprehensive evaluation of the general performance. However, the mean of these results is emphasized in the text to facilitate comparisons and discussions. These tables show the performance of models using only EEG signals, only EOG channels, both EEG and EOG channels together, and a reduced set of EEG signals (‘C3′, ’Cz’, and ’C4′) combined with EOG channels.

This current study reveals that EOG channels carry valuable information related to neural activity, rather than merely contributing noise. The results indicate that EOG channels can be used in EEG classification tasks, as shown in Table 3 and Table 4. For dataset 1, the accuracy with three EOG + two EEG channels is 83%, compared to 69.3% with three EEG channels. Similarly, for dataset 2, the accuracy with two EOG + three EEG channels is 61%, compared to 54.8% with three EEG channels.

The results also indicate that using a reduced number of EEG channels, along with EOG channels, provides similar or even better performance compared to using all available EEG channels. In Table 3, for dataset 1, the combination of three EEG and three EOG channels (a total of six channels) yields an accuracy of 83%, which is nearly identical to the accuracy of 84% achieved using all 22 EEG channels. In Table 4, for dataset 2, the combination of three EEG and two EOG channels (a total of five channels) results in an accuracy of 61%, surpassing the 57.4% accuracy obtained using all 60 EEG channels.

As previously mentioned, performance tends to decrease as the number of classes increases, making it more challenging to reduce the number of channels for high-class motor imagery signals. Table 5 shows that most studies in the literature focus on only two classes, highlighting the difficulty of channel reduction as the number of classes grows. To address this issue, multi-class datasets are selected, recognizing that most existing studies typically involve no more than four classes. The Weibo dataset is specifically chosen because it contains seven classes, aiming to provide a more comprehensive solution for BCI systems.

In Table 5, it can be seen that the BCI Competition IV Dataset IIa, a frequently preferred multi-class dataset in the literature, is also used in this study. Some of these studies selected only two out of the four classes to apply channel reduction. In comparison, this study achieved the most substantial channel reduction while evaluating all classes in a multi-class scenario and achieved the best performance with the same dataset. This improvement can be attributed to the addition of EOG channels, which aids in both noise reduction and providing information related to neural activity. Additionally, the proposed deep learning system demonstrates effective performance for motor imagery signals.

In this study, the deep learning model’s training and testing were performed using CUDA on an NVIDIA GeForce RTX 3050 Laptop GPU, supported by an AMD Ryzen 5 6600H CPU. This configuration leveraged the computational power of GPU acceleration, significantly enhancing processing speed and performance. The proposed deep learning model was trained using the Adam optimizer, set with a learning rate of 0.001, and employed Cross-Entropy loss as the loss function. Training proceeded for 500 epochs, with peak test accuracy for each configuration being carefully documented. Random training and test splitting were performed five times, with the results averaged and presented in the tables. For data handling, the MOABB [46] library (version 1.0.0) was used to load the EEG data, and various preprocessing techniques were applied with the MNE-Python [47,48] library (version 1.6.0).

## 4. Discussions

Following the idea provided in Section 3, the performance of all channels was analyzed using both filtering and wrapping-based methods. The analysis showed that EOG channels often achieved higher importance scores than EEG channels when assessed with techniques like mutual information, CSP, permutation importance, channel attention mechanism, and random search. These results highlight the effectiveness of EOG channels, which can provide valuable information, especially in combination with EEG signals. Table 3 and Table 4 further demonstrate that EOG channels are effective even when used independently of EEG channels, suggesting they carry significant information related to neural activity in motor imagery tasks.

In Figure 5 and Figure 6, the mutual information values for dataset 1 and dataset 2 indicate that EEG channels located near EOG channels tend to have higher values, implying potential overlapping information. However, this overlap could stem from either eye movement noise or valuable neural activity data or both. It is also believed that EOG channels may capture valuable information distinct from EEG signals, as suggested by the low mutual information values between VEOG and all EEG channels in dataset 2. Although VEOG shows low mutual information, EOG channels have demonstrated the ability to classify EEG signals, as shown in Table 4. This indicates that EOG channels may provide useful information such as the EEG signals. Of course, it is possible that one EOG channel, such as HEOG, carries useful data while another, like VEOG, does not, but further investigation is needed to clarify this distinction. While this study demonstrates the potential of EOG channels to capture neural activity, further research and additional datasets are required to determine the effects on the classification tasks of EEG signals.

## 5. Conclusions

In this study, the potential benefits of incorporating EOG channels into both the classification and channel reduction processes have been demonstrated. Unlike most studies in the literature, channel selection (or reduction) is applied in both subject-independent and multi-class scenarios. This approach makes the channel reduction and classification process more complex due to the variability across subjects and the increased number of classes. Despite these challenges, addressing channel reduction in such scenarios is crucial for developing more robust and adaptable BCI systems. Effective solutions for these scenarios enhance the flexibility and performance of BCIs, making them more practical for diverse applications. This study helps in developing BCI systems that are more generalizable and better suited for real-world use.

In future research, we aim to investigate the potential of each EOG channel individually and determine whether EOG channels capture similar neural activity to EEG channels or offer distinct information. To achieve this, we will utilize additional datasets with different types of EOG channels. As such, we seek to gain a deeper understanding of the unique contributions of EOG channels and their potential benefits for enhancing BCI systems.

## Figures and Tables

**Table 1:** Frequency range and cognitive states for each frequency band of EEG signals.

Frequency Bands	Frequency Range	Cognitive States
Delta	0–4 Hz	Sleep
Theta	4–7 Hz	Deep relaxation and meditation
Alpha	8–12 Hz	Relax, calm
Beta	12–30 Hz	Awake, normal alert

**Table 2:** Best channels for four different methods applied to dataset 1 and dataset 2.

Methods	Best Channels for Dataset 1	Best Channels for Dataset 2
CSP	CSP0: **EOG2, EOG0**	CSP0: EEG channels
	CSP1: EEG channels	CSP1: EEG channels
	CSP2: EEG channels	CSP2: **HEOG, VEOG**
	CSP3: EEG channels	CSP3: EEG channels
	CSP4: **EOG2, EOG1, EOG0**	CSP4: EEG channels
Permutation Importance	EEG7, EEG21, EEG18, EEG17, **EOG2**,…	**VEOG**, EEG25, EEG8, EEG29, EEG27, **HEOG**,…
Channel Attention	**EOG2, EOG0**, EEG18, EEG7,..	**VEOG**, EEG27, EEG35,…
Random Search	**EOG2, EOG0, EOG1**, EEG21,…	

**Table 3:** Accuracy results for dataset 1 using EEG only, EOG only, both EEG and EOG together, and a reduced set of EEG and EOG channels combined.

Subjects	22 EEG (%)	22 EEG + 3 EOG (%)	3 EOG (%)	3 EEG (%)	3 EEG + 3 EOG (%)
S1	81.8	85.3	62	65.5	79.3
S2	65.5	75.8	63.7	48.2	71.5
S3	91.3	89.6	51.7	82.7	83.6
S4	81	86.2	62.9	59.4	71.5
S5	83.6	95.6	94.8	69.8	95.6
S6	77.5	80.1	62.9	55.1	71.5
S7	92.2	91.3	90.5	85.3	92.2
S8	90.5	91.3	86.2	76.7	91.3
S9	93.1	96.5	75.8	81	90.5
Mean	84	87.9	72.2	69.3	**83**
Median	83.6	89.6	63.7	69.8	**83.6**
Standard Deviation	8.46	6.46	14.23	12.41	**9.3**

**Table 4:** Accuracy results for dataset 2 using EEG only, EOG only, both EEG and EOG together, and a reduced set of EEG and EOG channels combined.

Subjects	60 EEG (%)	60 EEG + 2 EOG (%)	2 EOG (%)	3 EEG (%)	3 EEG + 2 EOG (%)
S1	35.7	44.6	51.7	45.5	58
S2	47.3	57.1	49.1	41.9	56.2
S3	40.1	43.7	31.2	41	41
S4	66.9	79.4	62.5	62.5	67.8
S5	64.2	68.7	58.9	73.2	73.2
S6	45	48	50	44	42
S7	64.2	70.5	48.2	52.6	53.5
S8	66.9	64.2	59.8	73.2	73.2
S9	63.3	62.5	58.9	58.9	66
S10	81.2	87.5	81.2	55.3	79.4
Mean	57.4	62.6	55.1	54.8	**61**
Median	63.75	63.35	55.3	53.95	**62**
Standard Deviation	13.784	13.89	12.16	11.48	**12.47**

**Table 5:** Comparisons of studies involving electrode reduction processes.

Studies	# Subjects	Mean Classification Accuracy (%)	# Classes	# Electrodes	Dataset
In [10]	7	83.6	2	17	BCI IV-1
In [12]	5	74.5	2	~33.6	BCI III-4a
	3	85.6	2	~39.3	BCI III-3a
In [17]	5	75.1–98.5	2	16–47	BCI III-4a
In [18]	5	70.0–96.7	2	22–41	BCI III-4a
	7	70.0–90.0	2	17–28	BCI IV-1
In [20]	3	77.8	2	11–51	BCI III-3a
	5	86.9	2	35–114	BCI III-4a
	4	84.4	2	12–51	BCI IV-1
In [21]	3	91.9	2	13–30	BCI III-3a
	5	87.4	2	14–67	BCI III-4a
	4	81.6	2	3–46	BCI IV-1
In [22]	5	88.6	2	7–12	BCI III-4a
	4	84.4	2	6–10	BCI IV-1
In [29]	9	69	4	8	BCI IV-2a
In [28]	5	88	4	16	BCI III-4a
	9	75	4	17	BCI IV-2a
In [26]	5	89.3	2	depends on subjects	BCI III-4a
	3	84.1	2	depends on subjects	BCI IV-1
In [27]	5	90.2	2	depends on subjects	BCI III-4a
	4	82.8	2	depends on subjects	BCI IV-1
In this study	9	**83**	4	6	BCI IV-2a
	10	**61**	7	5	Weibo

## Data Availability

The datasets analyzed during this study are publicly available through the MOABB (Mother of All BCI Benchmarks) [46] library. MOABB provides standardized access to multiple EEG datasets, including the BCI Competition IV Dataset IIa and the Weibo dataset, which were used in this study. Further details about these datasets and how to access them can be found in the MOABB documentation. No new data were created during this study.

## References

[1] Sun L., Feng Z., Chen B., Lu N. (2018). A contralateral channel guided model for EEG based motor imagery classification. Biomed. Signal Process. Control..

[2] Iqbal M.A., Rahman M.M., Muhtasim S., Uddin Shubha S.E., Hasan M. Effect of EOG Artifact Removal on EEG Motor-Imagery Classification. Proceedings of the 2022 25th International Conference on Computer and Information Technology, ICCIT 2022.

[3] Wang D., Miao D., Blohm G. (2012). Multi-class motor imagery EEG decoding for brain-computer interfaces. Front. Neurosci..

[4] Chaisaen R., Autthasan P., Mingchinda N., Leelaarporn P., Kunaseth, Tammajarung S. (2020). Decoding EEG Rhythms during Action Observation, Motor Imagery, and Execution for Standing and Sitting. IEEE Sens. J..

[5] Jiang J., Zhou Z., Yin E., Yu Y., Hu D. (2014). Hybrid Brain-Computer Interface (BCI) based on the EEG and EOG signals. Bio-Med. Mater. Eng..

[6] Mannan M.M.N., Kamran M.A., Kang S., Jeong M.Y. (2018). Effect of EOG signal filtering on the removal of ocular artifacts and EEG-based brain-computer interface: A comprehensive study. Complexity.

[7] Croft R.J., Barry R.J. (2000). Removal of ocular artifact from the EEG: A review. Neurophysiol. Clin.

[8] Özkahraman A., Ölmez T., Dokur Z., Mylonas P., Kardaras D., Caro J. (2024). Impact of Noise Elimination Methods on Classification Performance in Motor Imagery EEG. Novel and Intelligent Digital Systems: Proceedings of the 4th International Conference (NiDS 2024).

[9] Özkahraman A., Ölmez T., Dokur Z. Determination of the Common Electrodes for Users and Increasing the Classification Accuracy of Motor Imagery EEG. Neural Comput. Appl..

[10] Qiu Z., Jin J., Zhang Y., Wang X. (2016). Generic Channels Selection in Motor Imagery-Based BCI. Advances in Cognitive Neurodynamics.

[11] Kirar J.S., Agrawal R.K. (2019). A combination of spectral graph theory and quantum genetic algorithm to find relevant set of electrodes for motor imagery classification. Appl. Soft Comput..

[12] Gaur P., McCreadie K., Pachori R.B., Wang H., Prasad G. (2021). An automatic subject specific channel selection method for enhancing motor imagery classification in EEG-BCI using correlation. Biomed. Signal Process. Control.

[13] Atyabi A., Luerssen M., Fitzgibbon S., Powers D.M.W. Evolutionary feature selection and electrode reduction for EEG classification. Proceedings of the 2012 IEEE Congress on Evolutionary Computation.

[14] Feng J.K., Jin J., Daly I., Zhou J., Niu Y., Wang X., Cichocki A. (2019). An Optimized Channel Selection Method Based on Multifrequency CSP-Rank for Motor Imagery-Based BCI System. Comput. Intell. Neurosci..

[15] Arvaneh M., Guan C., Ang K.K., Quek C. (2011). Optimizing the channel selection and classification accuracy in EEG-based BCI. IEEE Trans. Biomed. Eng..

[16] Shenoy H.V., Vinod A.P. An iterative optimization technique for robust channel selection in motor imagery based brain computer interface. Proceedings of the IEEE International Conference on Systems, Man and Cybernetics.

[17] He L., Hu Y., Li Y., Li D. (2013). Channel selection by Rayleigh coefficient maximization based genetic algorithm for classifying single-trial motor imagery EEG. Neurocomputing.

[18] Shi B., Wang Q., Yin S., Yue Z., Huai Y., Wang J. (2021). A binary harmony search algorithm as channel selection method for motor imagery-based BCI. Neurocomputing.

[19] Yang J., Singh H., Hines E.L., Schlaghecken F., Iliescu D.D., Leeson M.S., Stocks N.G. (2012). Channel selection and classification of electroencephalogram signals: An artificial neural network and genetic algorithm-based approach. Artif. Intell. Med..

[20] Jin J., Liu C., Daly I., Miao Y., Li S., Wang X., Cichocki A. (2020). Bispectrum-Based Channel Selection for Motor Imagery Based Brain-Computer Interfacing. IEEE Trans. Neural Syst. Rehabil. Eng..

[21] Jin J., Miao Y., Daly I., Zuo C., Hu D., Cichocki A. (2019). Correlation-based channel selection and regularized feature optimization for MI-based BCI. Neural Netw..

[22] Park Y., Chung W. (2020). Optimal Channel Selection Using Correlation Coefficient for CSP Based EEG Classification. IEEE Access.

[23] Zhou A., Zhang L., Yuan X., Li C. (2023). A signal prediction-based method for motor imagery EEG classification. Biomed. Signal Process. Control..

[24] Lee H.-C., Lee C.-H. (2023). Generalized Optimal EEG Channels Selection for Motor Imagery Brain-Computer Interface. IEEE Sens. J..

[25] Pawan, Dhiman R. (2022). Electroencephalogram channel selection based on pearson correlation coefficient for motor imagery-brain-computer interface. Meas. Sens..

[26] Meng M., Dong Z., Gao Y., She Q. (2022). Optimal channel and frequency band-based feature selection for motor imagery electroencephalogram classification. Int. J. Imaging Syst. Technol..

[27] Al Shiam A., Hassan K.M., Islam R., Almassri A.M.M., Wagatsuma H., Molla K.I. (2024). Motor imagery classification using effective channel selection of multichannel EEG. Brain Sci..

[28] Mahamune R., Laskar S.H. (2022). An automatic channel selection method based on the standard deviation of wavelet coefficients for motor imagery based brain–computer interfacing. Int. J. Imaging Syst. Technol..

[29] Tong L., Qian Y., Peng L., Wang C., Hou Z.-G. (2023). A learnable EEG channel selection method for MI-BCI using efficient channel attention. Front. Neurosci..

[30] Liang W., Jin J., Daly I., Sun H., Wang X., Cichocki A. (2022). Novel channel selection model based on graph convolutional network for motor imagery. Cogn. Neurodynamics.

[31] Amiri H.K., Zarei M., Daliri M.R. (2024). Motor imagery electroencephalography channel selection based on deep learning: A shallow convolutional neural network. Eng. Appl. Artif. Intell..

[32] Pudjihartono N., Fadason T., Kempa-Liehr A.W., O’Sullivan J.M. (2022). A Review of Feature Selection Methods for Machine Learning-Based Disease Risk Prediction. Front. Bioinform..

[33] Xia Y., Dong J., Li D., Li K., Nan J., Xu R. (2023). An Adaptive Channel Selection and Graph ResNet Based Algorithm for Motor Imagery Classification. Int. J. Adv. Comput. Sci. Appl..

[34] Tiwari A. (2023). A logistic binary Jaya optimization-based channel selection scheme for motor-imagery classification in brain-computer interface. Expert Syst. Appl..

[35] Dokur Z., Olmez T. (2021). Classification of motor imagery electroencephalogram signals by using a divergence based convolutional neural network. Appl. Soft Comput..

[36] Korhan N., Olmez T., Dokur Z. (2022). Generating ten BCI commands using four simple motor imageries and classification by divergence-based DNN. Neural Comput. Appl..

[37] Lawhern V.J., Solon A.J., Waytowich N.R., Gordon S.M., Hung C.P., Lance B.J. (2018). EEGNet: A compact convolutional neural network for EEG-based brain-computer interfaces. J. Neural Eng..

[38] Sabour S., Frosst N., Hinton G.E. (2017). Dynamic routing between capsules. Adv. Neural Inf. Process. Syst. arXiv.

[39] Ha K.-W., Jeong J.-W. (2019). Motor imagery EEG classification using capsule networks. Sensors.

[40] (2008). BCI Competitions IV-2a. https://www.bbci.de/competition/iv/.

[41] Brunner C., Leeb R., Müller-Putz G., Schlögl A., Pfurtscheller G. BCI Competition 2008—Graz Data Set A. Institute for Knowledge Discovery. Graz University of Technology, Graz, Austria. https://www.bbci.de/competition/iv/desc_2a.pdf.

[42] Yi W., Qiu S., Wang K., Qi H., Zhang L., Zhou P., He F., Ming D. (2014). Evaluation of EEG oscillatory patterns and cognitive process during simple and compound limb motor imagery. PLoS ONE.

[43] Cohen M.X. (2022). A tutorial on generalized eigendecomposition for denoising, contrast enhancement, and dimension reduction in multichannel electrophysiology. NeuroImage.

[44] Ghorbanzade G., Nabizadeh-ShahreBabak Z., Samavi S., Karimi N., Emami A., Khadivi P. (2020). Selection of proper EEG channels for subject intention classification using deep learning. arXiv.

[45] Haufe S., Meinecke F., Görgen K., Dähne S., Haynes J.-D., Blankertz B., Bießmann F. (2014). On the interpretation of weight vectors of linear models in multivariate neuroimaging. NeuroImage.

[46] Aristimunha B., Carrara I., Guetschel P., Sedlar S., Rodrigues P., Sosulski J., Narayanan D., Bjareholt E., Quentin B., Schirrmeister R.T. (2023). Mother of all BCI Benchmarks (MOABB). https://zenodo.org/records/13784463.

[47] Larson E. (2023). MNE-Python. Zenodo.

[48] Gramfort A., Luessi M., Larson E., Engemann D.A., Strohmeier D., Brodbeck C., Goj R., Jas M., Brooks T., Parkkonen L. (2013). MEG and EEG data analysis with MNE-Python. Front. Neurosci..

